# Node Classification Method Based on Hierarchical Hypergraph Neural Network

**DOI:** 10.3390/s24237655

**Published:** 2024-11-29

**Authors:** Feng Xu, Wanyue Xiong, Zizhu Fan, Licheng Sun

**Affiliations:** 1 School of Electrical and Automation Engineering, East China Jiaotong University, Nanchang 330013, China; 2022029081100008@ecjtu.edu.cn (F.X.); 2021088085400002@ecjtu.edu.cn (W.X.); 2 School of Mechanical and Electrical Engineering, Quzhou College of Technology, Quzhou 324000, China; 3 College of Computer Science and Technology, Shanghai University of Electric Power, Shanghai 200090, China; 4 Mechanical and Electrical Room, Quzhou Special Equipment Inspection & Testing Research Institute, Quzhou 324000, China; amenslc@163.com

**Keywords:** hypergraph neural networks, hierarchical representations, Nnode classification

## Abstract

Hypergraph neural networks have gained widespread attention due to their effectiveness in handling graph-structured data with complex relationships and multi-dimensional interactions. However, existing hypergraph neural network models mainly rely on planar message-passing mechanisms, which have limitations: (i) low efficiency in encoding long-distance information; (ii) underutilization of high-order neighborhood features, aggregating information only on the edges of the original graph. This paper proposes an innovative hierarchical hypergraph neural network (HCHG) to address these issues. The HCHG combines the high-order relationship-capturing capability of hypergraphs, uses the Louvain community detection algorithm to identify community structures within the network, and constructs hypergraphs layer by layer. In the bottom-level hypergraph, the model establishes high-order relationships through direct neighbor nodes, while in the top-level hypergraph, it captures global relationships between aggregated communities. Through three hierarchical message-passing mechanisms, the HCHG effectively integrates local and global information, enhancing the multi-resolution representation ability of node representations and significantly improving performance in node classification tasks. In addition, the model performs excellently in handling 3D multi-view datasets. Such datasets can be created by capturing 3D shapes and geometric features through sensors or by manual modeling, providing extensive application scenarios for analyzing three-dimensional shapes and complex geometric structures. Theoretical analysis and experimental results show that the HCHG outperforms traditional hypergraph neural networks in complex networks.

## 1. Introduction

Graph neural networks (GNNs) have garnered significant attention recently, emerging as powerful tools for processing graph-structured data. They are widely applied in various domains such as social networks [1,2], Photogrammetry [3,4], 3D object classification [5,6], the Internet of Things [7], and bioinformatics [8,9]. GNNs can effectively capture local relationships between nodes within a graph by aggregating information from neighboring nodes, enabling tasks such as node classification and link prediction [10,11]. For instance, in 3D object classification, GNNs utilize point cloud and depth data from sensors like LiDAR and RGB-D cameras, leveraging spatial relationships among nodes to enhance classification accuracy by capturing geometric features, especially in complex scenes. Classic GNN models, including GCN [12], GAT [13], and GraphSAGE [8], have achieved substantial advances in representation learning for graph data. However, these models exhibit limitations when handling complex graph structures and long-range dependencies among nodes.

Specifically, the flat message-passing mechanism of traditional GNNs makes it difficult to capture relationships between distant nodes effectively [14,15]. Furthermore, existing graph structures are often overly simplistic, primarily designed for binary relationships, which limits their ability to express multi-relational interactions fully. This directly results in traditional graph neural networks performing poorly in capturing global and local graph information, affecting classification effectiveness. Lastly, GNNs face memory and GPU memory constraints when handling complex graphs, making it challenging to scale to practical applications such as community detection [8].

The introduction of hypergraph structures presents a new approach to addressing these issues. Hypergraphs can capture high-order relationships among multiple nodes, transcending superficial binary relationships, thus better representing multilateral interactions in complex networks [16]. For example, hypergraphs are embedded into a low-dimensional space for clustering analysis, revealing the underlying group structures within the data. However, traditional hypergraph structures may have limitations when handling dynamically changing datasets, as they cannot adapt to rapidly evolving relationships, leading to delayed classification results [17]. At the same time, although hyperedge convolution layers can learn higher-order relationships in complex data, their high computational complexity affects the practicality of the model [18].

The hierarchical mechanism offers a new approach to addressing long-range dependencies and expressing hierarchical information. The hierarchical message-passing mechanism, which progressively aggregates local and global information, can effectively enhance the robustness and expressiveness of node representations [19]. For instance, hierarchical graph pooling methods, such as G-U-Net and DiffPool, have significantly improved in graph classification tasks [20,21]. Although some studies have combined hierarchical structures with graph learning models, there remains a lack of research on integrating hierarchical mechanisms with hypergraph neural networks for node classification.

To address key challenges in traditional GNNs and hypergraph neural networks, we introduce the Hierarchical Hypergraph Neural Network (HCHG). While GNNs struggle with long-range dependencies and global context, hypergraph neural networks excel at capturing higher-order relationships but are computationally expensive. HCHG constructs hypergraphs layer by layer, with the first layer capturing local relationships and subsequent layers aggregating community nodes to model global relationships, enhancing the model’s ability to represent local and global interactions. Additionally, HCHG uses a multi-layer message-passing mechanism, including bottom-up, lateral, and top-down flows, which strengthens node representations and reduces the computational burden, efficiently handling complex networks.

The main contributions of this paper are as follows:

1. We propose the HCHG model. This novel approach combines hierarchical structures with hypergraph neural networks to effectively capture local and global relationships in node classification and link prediction tasks, improving performance on complex graphs.
2. The HCHG model introduces a hierarchical construction method, using the Louvain community detection algorithm to build higher-order relationship networks, enhancing the model’s ability to represent complex network structures.
3. Our method performs excellently on six classification datasets and three link prediction datasets, achieving significant performance improvements across multiple tasks.

## 2. Related Work

Node classification is a core task in graph representation learning, aiming to predict a node’s category based on its structure and attributes. Although Graph Neural Networks (GNNs), such as GCNs [12] and GraphSAGE [8], have achieved significant progress in node classification tasks, they still face challenges in capturing long-range dependencies and handling sparse graph structures, which are critical for node classification in complex networks.

In recent years, hypergraph representation learning has provided new solutions by modeling higher-order relationships between nodes. For example, end-to-end hypergraph convolution [22] and Dynamic Hypergraph Neural Networks (DHGNN) [23] effectively capture complex node interactions. While simplifying computation, HyperGCN [24] may lose some high-order structural information. Furthermore, HyperSAGE [25] enhances generalization capabilities through an inductive message-passing mechanism. However, these methods still face limitations in integrating global and local information, particularly in dynamic and complex network scenarios. Researchers have introduced hierarchical structures into node classification tasks to enhance the capacity for multi-granularity semantic modeling. Methods such as DiffPool [21] and ASAP [19] significantly improve representation through node aggregation, but their applications in node-level tasks remain limited by the shortcomings of single-layer models.

We propose the HCHG, which leverages a multi-layer structure and diverse message-passing mechanisms to effectively integrate local and global information while enhancing adaptability and generalization. Experiments demonstrate that HCHG achieves outstanding performance across multiple node classification datasets, particularly excelling in modeling higher-order relationships and complex node interactions.

## 3. Motivation and Background

Given a hypergraph structure HG=(V,E,H), the objective is to construct a mapping function $F:HG\to Z\in R^{|V|\times d}$ that captures the features of node $v_{i}$ and its relationships within the hypergraph. The effectiveness of *F* will be evaluated through tasks such as node classification and link prediction.

In hypergraph neural networks, the node update mechanism differs from traditional Graph Neural Networks. A hypergraph consists of nodes and edges (hyperedges); each can connect multiple nodes. Below, we will detail the fundamental mechanism of node updates in hypergraph neural networks. Consider the hypergraph HG=(V,E,H), where *V* is the set of nodes, *E* is the set of edges, and *H* is the set of hyperedges. Each hyperedge $h_{i}\in H$ connects multiple nodes. The node update process in hypergraph neural networks typically involves the following two steps:

1. Message Aggregation

At layer *l*, the message aggregation for node *v* is completed through all hyperedges connected to *v*. The aggregation can be represented as follows:

(1) $$m_{agg}^{\left(l\right)}={\mathrm{Aggregate}}_{N}\left(\left\{W_{kv}^{\left(l\right)},h_{k}^{\left(l\right)}|k\in N\left(v\right)\right\}\right)$$

where ${\mathrm{Aggregate}}_{N}\left(\cdot \right)$ is a differentiable aggregation function, $W_{kv}^{\left(l\right)}$ is the association matrix between node *k* and node *v*, $h_{k}^{\left(l\right)}$ represents the features of node *k* at layer *l*, and N(v) is the set of neighbor nodes directly connected to node *v*.

2. Node Feature Update

Using the aggregated message $m_{agg}^{\left(l\right)}$, the feature of node *v* is updated as follows:

(2) $$\left\{\begin{array}{c} h_{v}^{\left(l\right)}=\mathrm{Combine}\left(m_{agg}^{\left(l\right)},m_{vgg}^{\left(l\right)}\right)\\ m_{vgg}^{\left(l\right)}={\mathrm{Aggregate}}_{I}\left(\left\{W_{kv}^{\left(l\right)}|k\in N\left(v\right)\right\}\right)h_{v}^{\left(l-1\right)} \end{array}\right.$$

where Combine(·) is a nonlinear fusion function, ${\mathrm{Aggregate}}_{I}\left(\cdot \right)$ is another aggregation function, and $h_{v}^{\left(l-1\right)}$ refers to the features of node *v* from the previous layer.

## 4. Hierarchical Hypergraph Neural Networks

**Figure 1:**
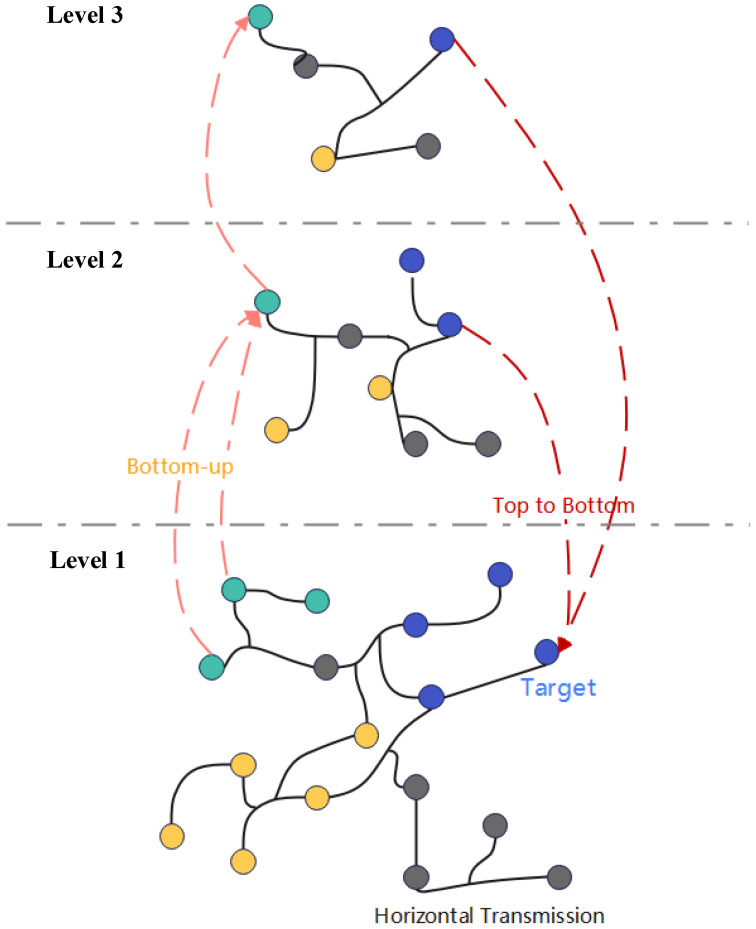
Hierarchical Hypergraph Neural Network Framework (Different colors represent different types of nodes).

We propose the HCHG framework to enable node representations to receive long-range messages and multi-granularity semantics through a hierarchical hypergraph structure. As illustrated in Figure 1, this framework first creates a progressively refined hierarchical structure, processing the input hypergraph in layers. Next, hypergraphs are constructed to facilitate message-passing between supernodes based on the connectivity among hierarchical nodes. To ensure adequate information flow, we design three message propagation mechanisms: bottom-up, intra-layer, and top-down. These mechanisms ensure information exchange both within the same level and across different levels. Finally, we train the model using task-specific loss functions and gradient descent algorithms, optimizing node representations and overall performance.

### 4.1. Hierarchical Structure Partitioning

For complex multi-node relational systems, we first represent the raw data as a graph G=(V,E), where *V* is the set of nodes representing different entities, and *E* is the set of edges depicting relationships between these entities. To capture the higher-order relationships among nodes more effectively, we introduce hyperedges *H*, defined as $h=\{v_{1},v_{2},\ldots ,v_{n}\}$, where $v_{i}\in V$. This constructs a hypergraph HG=(V,E,H).

On this basis, our study employs a hierarchical mechanism that simplifies the graph’s structure through layer-wise abstraction and aggregation. In each layer, we convert the node-set *V* into a higher-level set of supernodes by partitioning the nodes into communities. For instance, the supernodes in the first layer arise from the community partitioning of the original nodes. In contrast, subsequent layers’ supernodes are formed by combining the supernodes from the preceding layer. This approach allows the hierarchical structure to evolve progressively from the lower to the upper layers, facilitating higher-level analysis and modeling.

### 4.2. Hierarchical Hypergraph Construction

In constructing the hypergraph, the first layer hypergraph starts from the original. We utilize the Louvain community detection algorithm to identify communities within the node set *V* through modularity optimization. Each identified community $C_{i}\subseteq V$ forms a supernode, resulting in a supernode set $S_{1}=\left\{SC_{1}^{1},SC_{2}^{1},\ldots ,SC_{p}^{1}\right\}$, considered the first layer. Once the supernodes are established, we create edges between them. Suppose an edge exists between two communities, $C_{i}$ and $C_{j}$ (for example, through common original nodes or connecting hyperedges). In that case, we create an edge $e_{ij}$ between their corresponding supernodes $SC_{i}$ and $SC_{j}$.

We view the original nodes within each community as components of the hyperedges for hyperedge construction. Specifically, for the set of original nodes $V_{C_{i}}=\left\{v_{k}|v_{k}\in C_{i}\right\}$ within each community $C_{i}$, we define a hyperedge $h_{i}$ that includes all original nodes within community $C_{i}$, i.e., $h_{i}=V_{C_{i}}$. Thus, the set of hyperedges $H_{1}$ of the first layer hypergraph $HG_{1}$ consists of all hyperedges corresponding to the communities: $H_{1}=\left\{h_{i}|i=1,2,\ldots ,p\right\}$. Ultimately, the first layer hypergraph can be represented as $HG_{1}=\left(S_{1},E_{1},H_{1}\right)$, where $E_{1}$ is the set of edges between supernodes, and $H_{1}$ is the collection of hyperedges within $S_{1}$.

When generating the second layer hypergraph, we again apply the Louvain community detection algorithm to aggregate the first layer supernodes, forming the second layer supernode set $S_{2}=\left\{SC_{1}^{2},SC_{2}^{2},\ldots ,SC_{m}^{2}\right\}$. During this process, if there exists an edge between the second layer supernodes $SC_{k}^{2}$ and $SC_{l}^{2}$, we establish an edge $e_{kl}$ between them. Additionally, we construct hyperedges for the second layer supernodes, defining each hyperedge $h_{k}$ formed by the supernodes in community $C_{k}$. Specifically, each hyperedge $h_{k}$ in the second layer consists of all first layer supernodes $SC_{i}^{1}$ that belong to community $C_{k}$, i.e., $h_{k}=\left\{SC_{i}^{1}|SC_{i}^{1}\in C_{k}\right\}$. Ultimately, the second layer hypergraph is represented as $HG_{2}=\left(S_{2},E_{2},H_{2}\right)$, where $H_{2}$ is the collection of hyperedges within $S_{2}$.

### 4.3. Hierarchical Information Propagation

The hierarchical message-passing mechanism enhances node representations through long-range interaction and neighborhood aggregation. This mechanism does not interfere with the process of learning planar node representations, thereby effectively preserving the original information of the nodes. The hierarchical message-passing mechanism consists of the following three methods.

**Figure 2:**
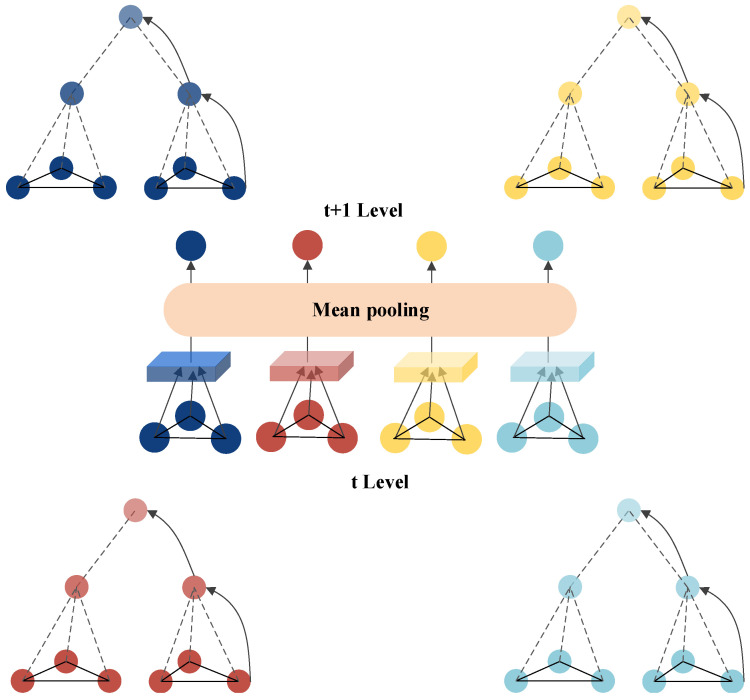
Schematic Diagram of Bottom-Up Propagation.

After obtaining the node representations of the (t−1)-th layer hypergraph $h_{s_{j}^{t-1}}^{\left(l\right)}$ using the node update mechanism, these representations are aggregated to update the supernode representations in the *t*-th layer. A schematic illustration is shown in Figure 2, with the mathematical expression for aggregation given by:

(3) $$a_{SC_{i}^{t}}^{\left(l\right)}=\frac{1}{|SC_{i}^{t}|+1}\left(\sum_{s_{j}^{t-1}\in SC_{i}^{t}}h_{s_{j}^{t-1}}^{\left(l\right)}+h_{SC_{i}^{t}}^{\left(l-1\right)}\right)$$

where $SC_{i}^{t}$ is the supernode in the *t*-th layer, $s_{j}^{t-1}$ represents the nodes belonging to $SC_{i}^{t}$ in the (t−1)-th layer, $|SC_{i}^{t}|$ is the number of nodes belonging to $SC_{i}^{t}$ in the (t−1)-th layer, and $h_{SC_{i}^{t}}^{\left(l-1\right)}$ represents the node representations of the supernode $SC_{i}^{t}$ in the *t*-th layer of the (l−1)-th layer.

### 4.4. Inter-Layer Propagation

**Figure 3:**
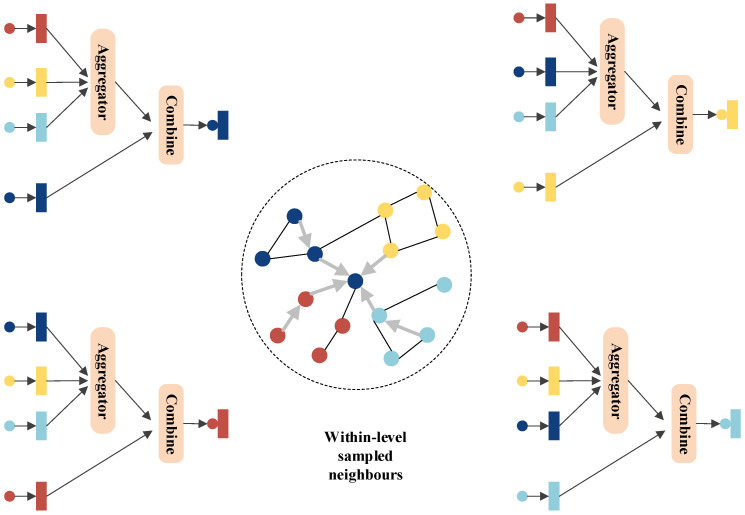
Schematic Diagram of Inter-Layer Propagation.

Inter-layer propagation primarily relies on hypergraph neural networks’ planar message-passing mechanism to aggregate neighboring information and update node representations within the same layer. A schematic illustration is shown in Figure 3. Based on the bottom-up propagation, the aggregation process of information from higher-layer supernodes is represented as follows:

(4) $$\left\{\begin{array}{c} m_{agg}^{\left(l\right)}={\mathrm{Aggregate}}_{N}\left(\left\{W_{kv}^{\left(l\right)},a_{u}^{\left(l\right)}|k\in N\left(v\right)\right\}\right)\\ m_{vgg}^{\left(l\right)}={\mathrm{Aggregate}}_{I}\left(\left\{W_{kv}^{\left(l\right)}|k\in N\left(v\right)\right\}\right)h_{v}^{\left(l-1\right)}\\ b_{v}^{\left(l\right)}=\mathrm{Combine}\left(m_{agg}^{\left(l\right)},m_{vgg}^{\left(l\right)}\right) \end{array}\right.$$

where $a_{u}^{\left(l\right)}$ is the representation of supernode *u* after bottom-up propagation, and $b_{v}^{\left(l\right)}$ is the updated feature representation of node *v* in layer *l*. The meanings of the remaining parameters are consistent with those in Section 3.

**Figure 4:**
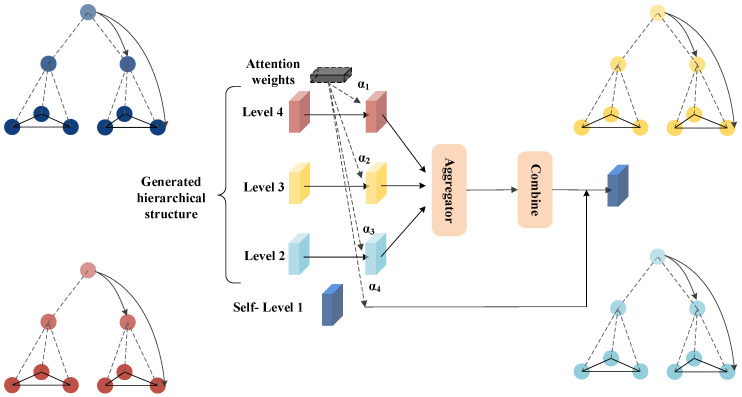
Schematic Diagram of Top-Down Propagation.

The node representations from the hypergraphs $\left\{G_{2},\ldots ,G_{T}\right\}$ are used to update the node representations in the original graph *G*. The importance of information from different layers varies depending on the specific task. Therefore, an attention mechanism proposed by Veličković et al. is employed to adaptively learn the weights of the information during the top-down integration process [26]. A schematic illustration is shown in Figure 4, represented as follows:

(5) $$h_{v}^{l}=\mathrm{ReLU}\left(\sum_{u\in N^{l}\left(v\right)}\alpha_{uv}^{l}W^{l}\cdot \mathrm{MEAN}\left(b_{u}^{l}\right)\right)$$

where $\alpha_{uv}^{l}$ is a trainable attention coefficient that represents the connection weight between nodes *v* and *u* across different layers. Here, $b_{u}$ is the bias term, MEAN denotes the element-wise average operation, and ReLU is the activation function. Ultimately, the node information representation from the last layer *L* is output using the following formula [27]:

(6) $$z_{v}=\sigma \left(\sum_{u\in N^{L}\left(v\right)}\alpha_{uv}^{L}W^{L}\cdot \mathrm{MEAN}\left(b_{u}^{L}\right)\right)$$

where σ is the Euclidean normalization function that adjusts values to the range of [0,1]. The final generated node representations $Z\in R^{n\times d}$ are used for classification, with each row $z_{v}\in Z$ representing the representation of a node *v*.

### 4.5. Model Training

In the experimental section, HCHG is applied to the training and prediction of semi-supervised node classification. The designed cross-entropy loss function is defined as follows:

(7) $$L=-\sum_{v\in V}y_{v}^{T}\log\left(\mathrm{Softmax}\left(z_{v}\right)\right)$$

where $y_{v}^{T}$ is the one-hot encoded label vector representing node *v*. The loss function *L* can be specifically modified based on different tasks.    

The computational complexity analysis presented in this paper consists of three main components: the hierarchical construction of graphs, the construction of hypergraphs, and the information propagation mechanism. The time complexity of the Louvain algorithm during the hierarchical construction process at each layer is $O(m_{t}\log n_{t})$. The complexity of hypergraph construction is $O(n_{t}+e_{t})$. The complexity of the information propagation mechanism is $O(n_{t}d_{t}+e_{t})$. Ultimately, the overall computational complexity can be expressed as:

(8) $$O\left(\sum_{t=1}^{T}\left(m_{t}\log n_{t}+n_{t}+e_{t}+n_{t}d_{t}\right)\right)$$

where *T* is the number of layers in the graph, and $m_{t}$, $n_{t}$, $e_{t}$, and $d_{t}$ represent the number of edges, nodes, hyperedges, and the average degree of nodes in layer *t*, respectively.

The following Algorithm 1 provides a brief summary of the construction process of the HCHG model.
**Algorithm 1:** Hierarchical Hypergraph Neural Network
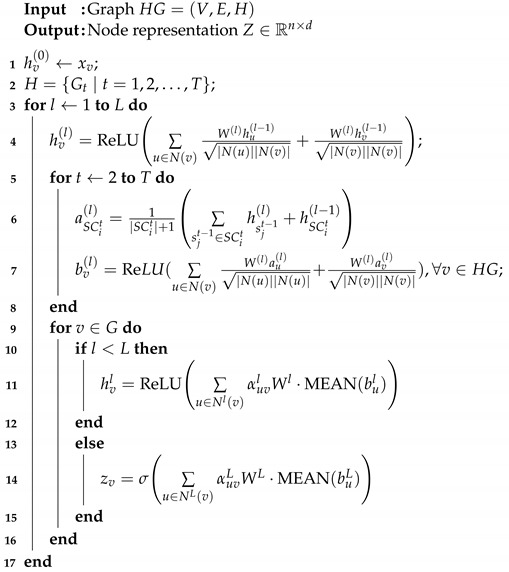


## 5. Experimental Analysis

### 5.1. Datasets

To verify the model’s overall performance, several commonly used datasets were used in the experiments, including graph-structured and multiview datasets. The graph-structured datasets contain rich node and edge relationships. In contrast, multi-view datasets can be generated by creating images from 3D models or using sensors to capture data from different angles, providing a comprehensive representation of the objects. Table 1 summarizes all the datasets used in the experiments.

1. Graph-structured Datasets

Cora and Citeseer are citation network datasets introduced by Sen et al. [28]. Cora consists of 2708 scientific publications and 5429 links. Each publication is represented as a node with a 1433-dimensional word vector as its feature. Citeseer consists of 3312 scientific publications and 4660 links, with each node having a 3703-dimensional word vector.

Pubmed, introduced by Namata et al. [29], includes 19,717 scientific publications about diabetes from the Pubmed database, divided into three classes. Each node is represented by a TF/IDF weighted word vector consisting of 500 words.

The Zoo dataset is downloaded from the UCI website. Each sample contains 17 Boolean attributes. Hyperedges are created for nodes with the same classification feature value.

Grid is a synthetic 2D grid graph representing a 20×20 grid with 400 nodes and no node features. This dataset is only for link prediction.

2. Multiview Datasets

The ModelNet40 dataset consists of 12,311 objects from 40 popular categories, split into training and test sets, with 9843 objects for training and 2468 objects for testing. NTU2012 (National Taiwan University (NTU) 3D Dataset) is a dataset from the computer vision/graphics field. It comprises 2012 3D shapes from 67 categories, including cars, chairs, chessboards, chips, clocks, cups, doors, frames, pens, plant leaves, etc. In the NTU2012 dataset, 80% of the data is used for training, and the remaining 20% is used for testing.

In the experiments, each 3D object is represented by extracted features. Two state-of-the-art shape representation methods, Multi-View Convolutional Neural Network (MVCNN) and Group-View Convolutional Neural Network (GVCNN), are adopted here. These two methods have shown satisfactory performance in representing 3D objects. Following the experimental settings of MVCNN and GVCNN, multiple views of each 3D object are generated.

### 5.2. Experimental Setup and Results

**Figure 5:**
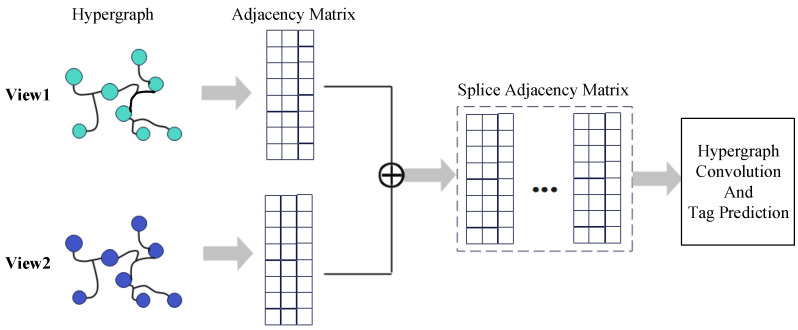
Multimodal Data Fusion.

Applying the HCHG model to the node classification task on the dataset, multiple averaged results are preserved in the experiments. For HGNN convolutional layers {256,128,64,32}, experiments are conducted with 2 or 3 convolutional layers and a $1\times 10^{-5}$ learning rate. The correlation between different views is modeled for the multiview data by generating a hypergraph *G*. For each view’s data, an adjacency matrix $H_{i}$ of the hypergraph is constructed based on the HGNN proposed by Feng et al. [22]. As shown in Figure 5, the adjacency matrices $H_{i}$ of different views are concatenated to construct the adjacency matrix *H* of the multiview hypergraph, thus creating a hypergraph structure with multiview features. Since the macro-level graph, $G_{T}$ of the macro-level layer is relatively small, during the experiment, we averaged the input label information by pooling $G_{T}$ and added the loss function separately to calculate the edge information in the *T* layer. The experimental results showed no impact on the data. The possible reason is that the macro-level layer contains very little information and has minimal influence on the target nodes after top-down propagation. Therefore, the small changes in the node connection mode in $G_{T}$ have a negligible effect on the results.

A comparison of the HCHG model with other graph neural network models for node classification was conducted. Experimental results were collected for eight models, including Hyper-Conv, HC-GNN [27], GCN [12], GAT [13], FastGCN [30], LADIES [31], and DNGNN [32], HJRL [33] on standard datasets, as shown in Table 2. For the link prediction, six models—GCN, GraphSAGE [8], GIN [14], G-U-Net [20], GXN [34], and HC-GNN—were compared, as shown in Table 3. The results with node and without node features were evaluated in the experiments with the Cora dataset. The results show that HCHG performs well in terms of node classification and link prediction accuracy.

For the multiview dataset (Table 4), a comparison was made between Hyper-Conv, LADIES, HGNN, HJRL, HGNN+ [35], and HC-HGNN. HCHG showed an improvement of approximately 6% on the NTU2012 dataset compared to other models, while HCHG and HC-GNN showed improvements on the ModelNet40 dataset. The superior performance of HCHG may be attributed to its hierarchical structure, which allows the model to capture the topological information of the graph, i.e., the message propagated from distant nodes in the graph. Moreover, the intermediate and macro-level semantics reflected in the hierarchical structure are encoded through bottom-up, intra-layer, and top-down propagation. On the ModelNet40 dataset, the HCHG model achieved an accuracy of 97%, surpassing other models such as Hyper-Conv, which attained 92%, demonstrating its adaptability to diverse data structures.

Additionally, with its ability to simultaneously aggregate information from multiple nodes, the hypergraph structure enables better capture of community structure information during the learning process. For example, on the NTU dataset, the HCHG model achieved an accuracy of 90%, a significant improvement compared to other models like HC-GNN, which achieved 85%.

The HCHG model demonstrates strong generalization capabilities when handling multimodal data, effectively adapting to various datasets. Its performance across diverse datasets, including Cora, Pubmed, Citeseer, Zoo, NTU, and ModelNet40, surpasses other models.

By integrating hypergraph structures and hierarchical information, the HCHG model can more effectively capture complex relationships surrounding nodes. In ablation experiments, the HCHG model consistently yielded favorable results across different numbers of node neighbors, confirming its adaptability to graphs of varying scales.

In summary, by combining hypergraph structures and hierarchical information, the HCHG model efficiently captures the intricate associations among multimodal data and achieves outstanding performance in node classification across various datasets.

**Figure 6:**
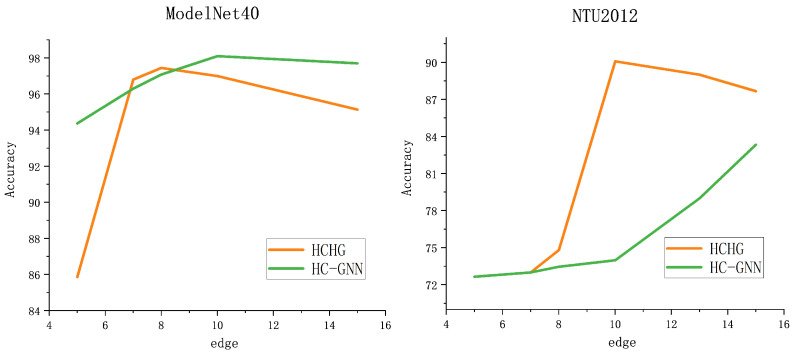
The number of neighboring node points affects the classification.

Finally, in the experiments, HC-GNN, which is constructed using GCN, was compared to HCHG to investigate the impact of the hypergraph structure on the experimental results. It was found that the effect varies for different datasets. Not all graph community structures are suitable for the hypergraph structure, and this needs to be considered in different problem scenarios. Table 5 presents the ablation experiments on the multiview data, comparing the classification results for the same nodes under various scenarios of single-view and multiview. The results from the three models in the experiment demonstrated that multiview data carries more information, which is beneficial for classification. Finally, a comparison was made on the number of neighboring nodes using the ModelNet40 and NTU2012 datasets. As shown in Figure 6, the different performances of the HC-GNN and the proposed HCHG model with varying numbers of neighboring nodes are displayed. It can be observed that the HCHG model achieves the best results even with fewer neighboring nodes, indicating that the hypergraph structure can aggregate neighbor information more quickly and accurately.

### 5.3. Visualization

**Figure 7:**
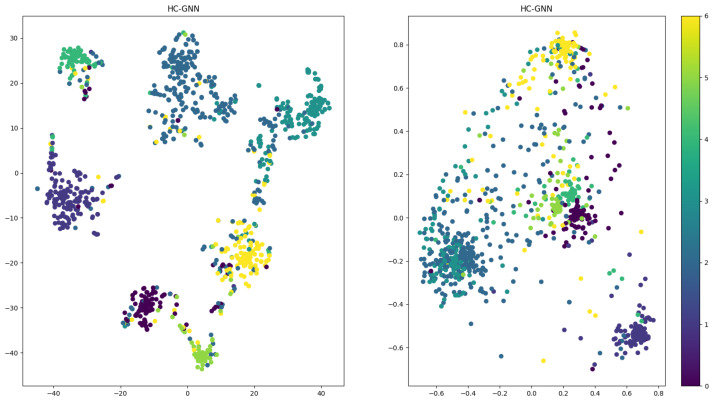

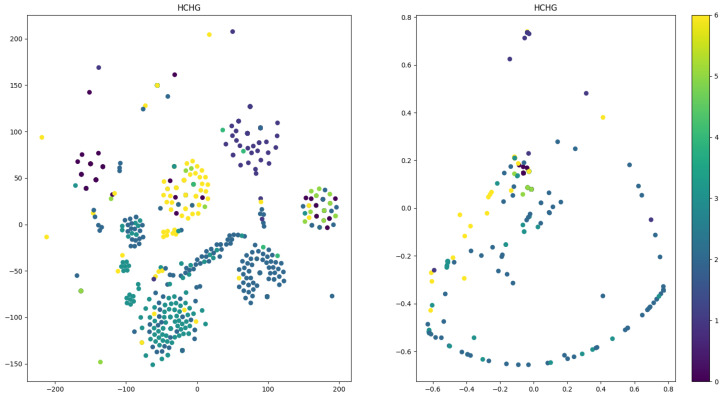
Visualization of the clustering results.

The core dataset was visualized to compare the learning abilities of graph-based and hypergraph-based methods intuitively. The t-SNE method was used to visualize the output of the last layer convolution. The results are shown in Figure 7. It can be seen from the results that compared to the graph-based method; the hypergraph-based HCHG method produces recognizable clusters, which qualitatively validates the effectiveness of the proposed method.

## 6. Conclusions

This paper proposes a novel Hierarchical message-passing Hypergraph Convolutional (HCHG) model that combines hypergraphs and hierarchical message-passing using a layered community detection algorithm. The HCHG model constructs a hierarchical structure of hypergraph neural networks and performs layered message-passing to handle multi-view data. The model structure of HCHG enables nodes to capture information-rich interactions from distant nodes effectively. Extensive experiments are conducted on five datasets, and the results are analyzed. The experiments demonstrate that HCHG performs excellently in graph-structured dataset classification and 3D model classification tasks. HCHG allows for different choices and customized designs of hierarchical structures, making it easily applicable to various task-specific data. In the future, our goal is to optimize the learning of hypergraph hierarchical structures further and extend the framework to handle complex multimodal data in real-life scenarios.

## 7. Limitations and Future Work

Despite the impressive performance of the HCHG model on multiple datasets, there are still some limitations. Future work will focus on improving the model’s computational efficiency, particularly its scalability in complex networks, and developing more efficient algorithms to reduce computational complexity. Additionally, we plan to optimize the model’s ability to handle complex social hierarchies and nested structural networks. Finally, we will conduct an in-depth analysis of how different community detection methods impact the generation of hierarchical structures, exploring their applicability and limitations in real-world scenarios. Overall, future research will further enhance the performance and broad applicability of the HCHG model.

## Figures and Tables

**Table 1:** Categorical Dataset.

Dataset	Nodes/Feature	Train/(val)/Test	Class
Cora	2708/1433	140/500/1000	7
Citeseer	3312/3703	140/500/1000	6
Pubmed	19,717/500	60/500/1000	3
Zoo	101/16	66/35	7
Grid	400	-	-
ModelNet40	12,311/(2048/4096)	9843/2468	44
NTU2012	2012/(2048/4096)	1640/372	67

**Table 2:** Average Test Accuracy (%) ± Standard Deviation for Node Classification Tasks.

Model (Author, Year)	Zoo	Cora	Pubmed	Citeseer
GCN [12]	60.0 ± 1.5	80.2 ± 0.9	77.9 ± 1.1	64.8 ± 1.4
GAT [13]	48.5 ± 1.2	77.2 ± 1.0	77.5 ± 0.8	62.0 ± 1.3
FastGCN [30]	37.8 ± 1.6	78.0 ± 0.8	74.4 ± 1.3	63.5 ± 1.5
LADIES [31]	37.8 ± 1.7	78.3 ± 0.7	76.8 ± 1.2	65.0 ± 1.1
Hyper-Conv [27]	93.1 ± 0.4	82.7 ± 0.5	78.4 ± 0.6	71.2 ± 0.7
LE [27]	97.0 ± 0.2	82.3 ± 0.4	78.7 ± 0.5	70.4 ± 0.6
HC-GNN [27]	85.7 ± 0.5	79.0 ± 0.6	78.7 ± 0.4	65.9 ± 1.0
HJRL [29]	96.3 ± 0.3	77.6 ± 0.5	77.3 ± 0.6	65.1 ± 1.2
HCHG (Ours)	97.1 ± 0.2	79.8 ± 0.6	79.4 ± 0.5	66.2 ± 1.0

**Table 3:** Average Test Accuracy (%) ± Standard Deviation for Link Prediction Tasks.

Model (Author, Year)	Grid	Cora-Feat	Cora-noFeat
GCN [12]	76.3 ± 1.2	86.9 ± 0.9	78.5 ± 1.1
GraphSAGE [8]	77.5 ± 1.1	87.0 ± 0.7	74.1 ± 1.3
GIN [14]	75.6 ± 1.0	86.2 ± 0.8	78.2 ± 1.2
G-U-Net [20]	70.1 ± 1.5	90.9 ± 0.6	77.2 ± 1.0
GXN [34]	64.2 ± 1.4	88.9 ± 0.8	78.1 ± 1.1
HC-GNN [27]	80.1 ± 1.3	89.4 ± 0.7	77.6 ± 1.0
HCHG (Ours)	87.8 ± 0.9	82.1 ± 1.2	78.5 ± 1.1

**Table 4:** Average Test Accuracy (%) ± Standard Deviation for Node Classification Tasks.

Model (Author, Year)	NTU2012	ModelNet40
Hyper-Conv [27]	79.4 ± 0.8	91.1 ± 1.2
LE [27]	83.2 ± 1.6	94.1 ± 1.3
HGNN [35]	84.2 ± 1.4	96.7 ± 1.2
HGNN+ [35]	84.2 ± 1.5	96.9 ± 1.1
HC-GNN [27]	83.3 ± 1.0	98.1 ± 0.8
HJRL [29]	86.1 ± 1.3	95.8 ± 1.4
HCHG (ours)	90.0 ± 1.1	97.4 ± 1.3

**Table 5:** Average Test Accuracy (%) ± Standard Deviation for Multi-view Data vs. Single-view Data Comparison.

View	HC-GNN [27]	HGNN [35]	HJRL [29]	HCHG (Ours)
NTU (mvcnn)	73.7 ± 1.1	69.8 ± 0.9	69.6 ± 1.2	70.7 ± 1.0
NTU (gvcnn)	69.7 ± 0.8	79.5 ± 0.7	80.3 ± 1.6	85.7 ± 1.2
NTU (mvc. and gvc.)	83.3 ± 1.0	84.2 ± 1.4	86.1 ± 1.3	90.0 ± 0.8
ModelNet40 (mvcnn)	98.1 ± 1.3	90.8 ± 1.0	92.3 ± 1.7	93.9 ± 1.1
ModelNet40 (gvcnn)	97.3 ± 1.1	92.8 ± 0.9	90.5 ± 1.4	81.5 ± 1.5
ModelNet40 (mvc. and gvc.)	98.1 ± 0.8	96.7 ± 1.2	95.8 ± 1.4	97.4 ± 1.3

## Data Availability

The data presented in this study are available in the article.

## References

[1] Min S., Gao Z., Peng J., Wang L., Qin K., Fang B. (2021). STGSN—A spatial–temporal graph neural network framework for time-evolving social networks. Knowl.-Based Syst..

[2] Dhelim S., Aung N., Ning H. (2020). Mining user interest based on personality-aware hybrid filtering in social networks. Knowl.-Based Syst..

[3] Leahy J., Jabari S. (2024). Enhancing Aerial Camera-LiDAR Registration through Combined LiDAR Feature Layers and Graph Neural Networks. Int. Arch. Photogramm. Remote Sens. Spat. Inf. Sci..

[4] Yuan W., Yuan X., Fan Z., Guo Z., Shi X., Gong J., Shibasaki R. (2021). Graph neural network based multi-feature fusion for building change detection. Int. Arch. Photogramm. Remote Sens. Spat. Inf. Sci..

[5] Shi W., Rajkumar R. Point-gnn: Graph neural network for 3d object detection in a point cloud. Proceedings of the IEEE/CVF Conference on Computer Vision and Pattern Recognition.

[6] Meraz M., Ansari M.A., Javed M., Chakraborty P. (2022). DC-GNN: Drop channel graph neural network for object classification and part segmentation in the point cloud. Int. J. Multimed. Inf. Retr..

[7] Wu Y., Dai H.N., Tang H. (2022). Graph neural networks for anomaly detection in industrial Internet of Things. IEEE Internet Things J..

[8] Hamilton W., Ying Z., Leskovec J. Inductive representation learning on large graphs. Proceedings of the International Conference on Advances in Neural Information Processing Systems (NeurIPS).

[9] Huang K., Xiao C., Glass L.M., Zitnik M., Sun J. (2020). SkipGNN: Predicting molecular interactions with skip-graph networks. Sci. Rep..

[10] Zitnik M., Agrawal M., Leskovec J. (2018). Modeling polypharmacy side effects with graph convolutional networks. Bioinformatics.

[11] Zhang M., Chen Y. Link prediction based on graph neural networks. Proceedings of the 2018 Annual Conference on Neural Information Processing Systems (NeurIPS).

[12] Kipf T.N., Welling M. Semi-supervised classification with graph convolutional networks. Proceedings of the International Conference on Learning Representations (ICLR).

[13] Veličković P., Cucurull G., Casanova A., Romero A., Liò P., Bengio Y. Graph attention networks. Proceedings of the International Conference on Learning Representations (ICLR).

[14] Xu K., Hu W., Leskovec J., Jegelka S. How powerful are graph neural networks? In Proceedings of the 2019 International Conference on Machine Learning (ICML), Long Beach, CA, USA, 9–15 June 2019.

[15] Min Y., Wenkel F., Wolf G. Scattering GCN: Overcoming oversmoothness in graph convolutional networks. Proceedings of the 2020 Annual Conference on Neural Information Processing Systems (NeurIPS).

[16] Perozzi B., Al-Rfou R., Skiena S. Deepwalk: Online learning of social representations. Proceedings of the 20th ACM SIGKDD International Conference on Knowledge Discovery and Data Mining.

[17] Zhou D., Huang J., Schölkopf B. Learning with hypergraphs: Clustering, classification, and embedding. Proceedings of the Advances in Neural Information Processing Systems.

[18] Feng Y., You H., Zhang Z., Ji R., Gao Y. Hypergraph neural networks. Proceedings of the AAAI Conference on Artificial Intelligence.

[19] Ranjan E., Sanyal S., Talukdar P.P. ASAP: Adaptive structure aware pooling for learning hierarchical graph representations. Proceedings of the 2020 AAAI Conference on Artificial Intelligence (AAAI).

[20] Gao H., Ji S. Graph U-Nets. Proceedings of the 2019 International Conference on Machine Learning (ICML).

[21] Ying R., You J., Morris C., Ren X., Hamilton W.L., Leskovec J. Hierarchical graph representation learning with differentiable pooling. Proceedings of the 2018 Annual Conference on Neural Information Processing Systems (NeurIPS).

[22] Bai S., Zhang F., Torr P.H. (2021). Hypergraph convolution and hypergraph attention. Pattern Recognit..

[23] Jiang J., Wei Y., Feng Y., Cao J., Gao Y. Dynamic hypergraph neural networks. Proceedings of the IJCAI.

[24] Yadati N., Nimishakavi M., Yadav P., Nitin V., Louis A., Talukdar P. Hypergcn: A new method for training graph convolutional networks on hypergraphs. Proceedings of the Advances in Neural Information Processing Systems.

[25] Arya D., Gupta D.K., Rudinac S., Worring M. (2020). Hypersage: Generalizing inductive representation learning on hypergraphs. arXiv.

[26] Ye Z., Zhao H., Zhang K., Zhu Y., Xiao Y. (2019). Tri-party deep network representation learning using inductive matrix completion. J. Cent. South Univ..

[27] Zhong Z., Li C.T., Pang J. (2023). Hierarchical message-passing graph neural networks. Data Min. Knowl. Discov..

[28] Sen P., Namata G., Bilgic M., Getoor L., Galligher B., Eliassi-Rad T. (2008). Collective classification in network data. AI Mag..

[29] Namata G., London B., Getoor L., Huang B. Query-driven active surveying for collective classification. Proceedings of the 2012 International Workshop on Mining and Learning with Graphs.

[30] Chen J., Ma T., Xiao C. (2018). FastGCN: Fast learning with graph convolutional networks via importance sampling. arXiv.

[31] Yang C., Wang R., Yao S., Abdelzaher T. (2020). Hypergraph learning with line expansion. arXiv.

[32] Sunil K.M. (2023). Feature Selection: Key to Enhance Node Classification with Graph Neural Networks). CAAI Trans. Intell. Technol..

[33] Yan Y., Chen Y., Wang S., Wu H., Cai R. Hypergraph Joint Representation Learning for Hypervertices and Hyperedges via Cross Expansion. Proceedings of the AAAI Conference on Artificial Intelligence.

[34] Li M., Chen S., Zhang Y., Tsang I.W. Graph cross networks with vertex infomax pooling. Proceedings of the 2020 Annual Conference on Neural Information Processing Systems (NeurIPS).

[35] Gao Y., Feng Y., Ji S., Ji R. (2023). HGNN+: General Hypergraph Neural Networks. IEEE Trans. Pattern Anal. Mach. Intell..

